# The Denture-Associated Oral Microbiome in Health and Stomatitis

**DOI:** 10.1128/mSphere.00215-16

**Published:** 2016-12-28

**Authors:** Baochen Shi, Tingxi Wu, Jeffrey McLean, Anna Edlund, Youngik Young, Xuesong He, Hongyang Lv, Xuedong Zhou, Wenyuan Shi, Huiying Li, Renate Lux

**Affiliations:** a Department of Molecular and Medical Pharmacology, Crump Institute for Molecular Imaging, David Geffen School of Medicine, University of Los Angeles at Los Angeles, Los Angeles, California, USA; b School of Dentistry, University of Los Angeles at Los Angeles, Los Angeles, California, USA; c Department of Periodontics, School of Dentistry, University of Washington, Seattle, Washington, USA; d Microbial and Environmental Genomics, J. Craig Venter Institute, La Jolla, California, USA; e West China School of Stomatology, Sichuan University, Chengdu, Sichuan, China; University of Michigan

**Keywords:** bacteria, denture, microbiome, stomatitis

## Abstract

While investigation of the microbiome on natural oral surfaces has generated a wealth of information, few studies have examined the microbial communities colonizing dentures and their relationship to oral health. To address this knowledge gap, we characterized the bacterial community associated with dentures and remaining teeth in healthy individuals and patients with denture stomatitis. The microbiome compositions of matched denture and tooth plaque samples of 10 healthy individuals and 9 stomatitis patients were determined by 16S rRNA gene pyrosequencing. The microbial communities colonizing dentures and remaining teeth in health and disease were very similar to each other. Matched denture and tooth samples from the same individuals shared a significantly higher percentage of identical phylotypes than random pairs of samples from different study participants. Despite these overall similarities, several bacterial phylotypes displayed discrete health- and stomatitis-associated denture colonization, while others were distinct in health and disease independently of the surface. Certain phylotypes exhibited differential colonization of dentures and teeth independently of denture health status. In conclusion, denture and natural tooth surfaces in health and stomatitis harbor similar bacterial communities. Individual-related rather than surface-specific factors play a significant role in the bacterial phylotype composition colonizing dentures and teeth. This individual-specific mutual influence on denture and tooth surface colonization could be an important factor in maintaining oral health in denture wearers. Discrete differences in colonization patterns for distinct genera and phylotypes warrant further studies regarding their potential involvement or utility as specific indicators of health and disease development in denture-wearing individuals.

**IMPORTANCE** Denture stomatitis is a prevalent inflammatory condition of the mucosal tissue in denture wearers that is triggered by microorganisms. While *Candida* has been extensively studied for its role in stomatitis etiology, the bacterial component largely remains to be investigated. Our data show that certain types of bacteria are significantly associated with denture health and disease. Furthermore, the bacterial communities residing on the teeth and dentures of the same person are similar to each other independently of the surface, and thus, denture health could impact the maintenance of remaining teeth and vice versa.

## Abstract (precis)

Denture stomatitis is a prevalent inflammatory condition of the mucosal tissue in denture wearers that is triggered by microorganisms. While *Candida* has been extensively studied for its role in stomatitis etiology, the bacterial component largely remains to be investigated. Our data show that certain types of bacteria are significantly associated with denture health and disease. Furthermore, the bacterial communities residing on the teeth and dentures of the same person are similar to each other independently of the surface, and thus, denture health could impact the maintenance of remaining teeth and vice versa.

## INTRODUCTION

Both oral health and the microbial inhabitants of the oral cavity, in particular, are increasingly being recognized for their role in overall human health and disease (1). Connections between oral microbial infections and numerous systemic disease conditions have been established (2), and oral biofilms have been suspected to serve as reservoirs for infectious disease agents (3). With its combination of soft tissue and hard surfaces, the oral cavity comprises a unique environment for microbial colonization. The combined efforts of a number of research groups have led to a comprehensive inventory of oral microorganisms (4–6). In addition to the natural surfaces of the oral cavity, microorganisms can effectively form biofilms on the artificial hard surfaces that are introduced as part of dental restoration. Biofilm formation on restorative materials positively correlates with higher surface roughness and surface free energy (7). Among the biofilms colonizing artificial hard surfaces, those forming on dental implants have attracted a lot of attention in research. Healthy implants harbor very distinct microbiotas compared to teeth (8), and depending on the study, different microbial species have been suggested to be involved in peri-implant disease development (9). In contrast, the bacterial communities colonizing dentures, another important artificial surface present in the oral cavity of a significant part of the population, largely remain to be investigated. Even though some research groups have evaluated the denture-associated microbiota (10–17), the majority of studies are focused on special aspects, including the specific groups of bacteria or the effect of cleaning agents.

Over 20% of the people over 65 years of age in the United States are missing all of their teeth. With the increasing proportion of elderly people in the population, this proportion is likely to rise (18, 19). The bacteria colonizing dentures comprise an important part of the human microbiome to be studied for their role in maintaining oral health in the elderly. Denture wearing has been associated with a number of microbial diseases, including denture-related mucosal tissue inflammation (denture stomatitis) (20) and malodor (21). Dentures are also suspected to serve as a reservoir for respiratory pathogens (3, 13) and thus lead to an increased risk of pulmonary infections (22). Despite the fact that microorganisms are the obvious suspects for most of the above denture-related diseases, little is known about their etiology. Most studies investigating denture-associated microorganisms focus on colonization with *Candida* sp., which is considered an important etiological agent for denture stomatitis (23), even though bacterial species have been implicated in this oral disease as well (11, 16, 24). Currently, only limited knowledge is available regarding the microbial composition of biofilms growing on dentures. Comparisons with the microbiota residing on natural oral surfaces to elucidate if and how the microorganisms colonizing the denture shape the microbiota of the oral cavity and vice versa remain to be performed. Very few studies have assessed bacterial denture colonization by using culture-independent clone library and checkerboard approaches (10, 14, 15, 17). To date, only one recent study has reported a next-generation sequencing analysis, which provided an initial predominantly class level analysis of the microbiota colonizing dentures, the respective mucosal surfaces, and selected remaining teeth (12). While this is an important step toward understanding the bacterial component of health and disease in denture wearers, a recent comprehensive evaluation of different oral sites at the oligotype level has highlighted the importance of resolving communities at more detailed taxonomic levels to better understand the ecological and functional diversity of the microbiota relevant to health and disease (25). This level of analysis is still missing for the denture-associated microbiota and the corresponding microbial communities on the remaining teeth of denture wearers.

In this study, we performed a comprehensive genus and species level 16S rRNA gene sequencing-based analysis of the microbial biofilms colonizing dentures. Matched samples from dentures and remaining teeth from healthy individuals and those with denture stomatitis but no other oral diseases were used to allow comparison of patient- and surface-related factors. We investigated if the biofilms present on these different surfaces are distinct, if health- and disease-associated biofilm communities can be distinguished, and if microbial communities present on the different surfaces in the same patients influence each other. Identification of relevant disease-associated factors and microorganisms will enhance the ability of dentists to develop more targeted approaches for the treatment of denture-associated diseases.

## RESULTS

### Patient characteristics, sample collection, and sequencing.

Samples were collected individually from the dentures and remaining teeth of 10 healthy denture wearers and 10 patients with denture stomatitis who were otherwise free of oral microbial diseases (see Materials and Methods for further details). We identified the taxonomic composition of the oral microbiota by 454 pyrosequencing hypervariable regions V1 to V3 of the 16S rRNA genes for a total of forty samples (one denture- and one tooth-derived sample per patient collected as described in Materials and Methods). We obtained a total of 106,894 reads with a mean read length of 444 bp after removal of low-quality and short sequences, as well as chimeric sequences. One of the samples derived from the remaining teeth of a stomatitis patient that yielded <1,500 reads was excluded from further analysis together with the matching denture sample because of a lack of sufficient sequencing depth. An average of 2,739 reads (range, 1,692 to 4,975) was analyzed for each sample, which provides sufficient sequencing depth to capture the overall diversity of the samples (see Fig. S1 in the supplemental material).

10.1128/mSphere.00215-16.1 Figure S1  Rarefaction curve of V1 to V3 16S rRNA gene sequences after application of the quality control and cleanup procedures described in Materials and Methods. Data are presented as mean values ± the SEM. Download Figure S1, TIF file, 0.2 MB. Copyright © 2016 Shi et al. 2016 Shi et al. This content is distributed under the terms of the Creative Commons Attribution 4.0 International license.

### The community structures of denture- and tooth-associated microbiomes in healthy and diseased denture wearers are very similar.

**FIG 1:**
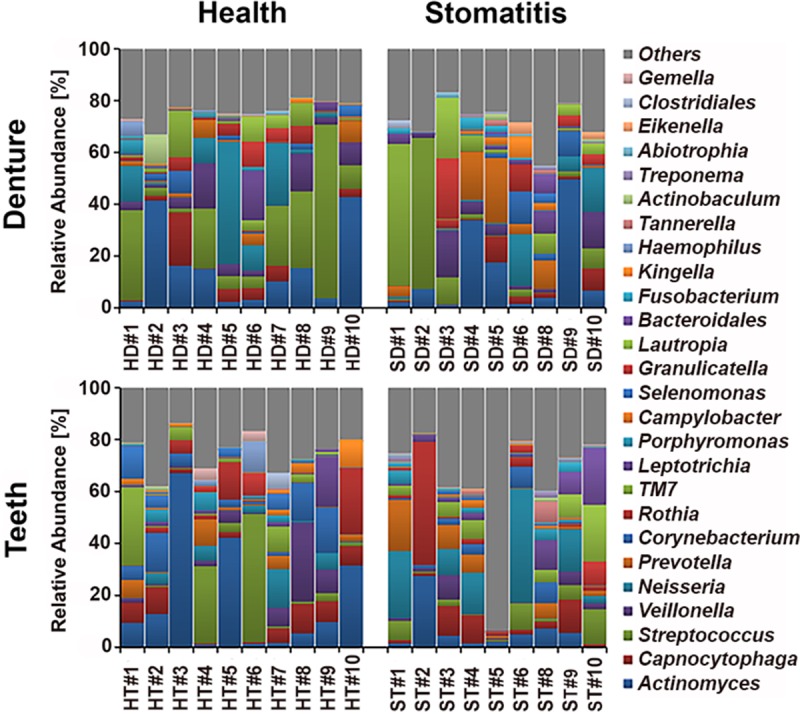
Genus level composition of denture- and tooth-associated microbiomes in health and stomatitis. The relative abundance at the genus level in denture (top) and tooth (bottom) samples collected from the same individuals are shown in the same order (H1 to H10, S1 to S6, and S8 to S10). The samples from healthy subjects are shown on the left, and those from stomatitis patients are shown on the right. HD, healthy denture; SD, stomatitis denture; HT, healthy remaining teeth; ST, stomatitis remaining teeth. Genera present with a >1% relative abundance in at least two patient samples were included in the analysis and are listed on the right with the respective color coding. Genera present at a <1% relative abundance or in only one patient sample were combined in the category “Others.”

**FIG 2:**
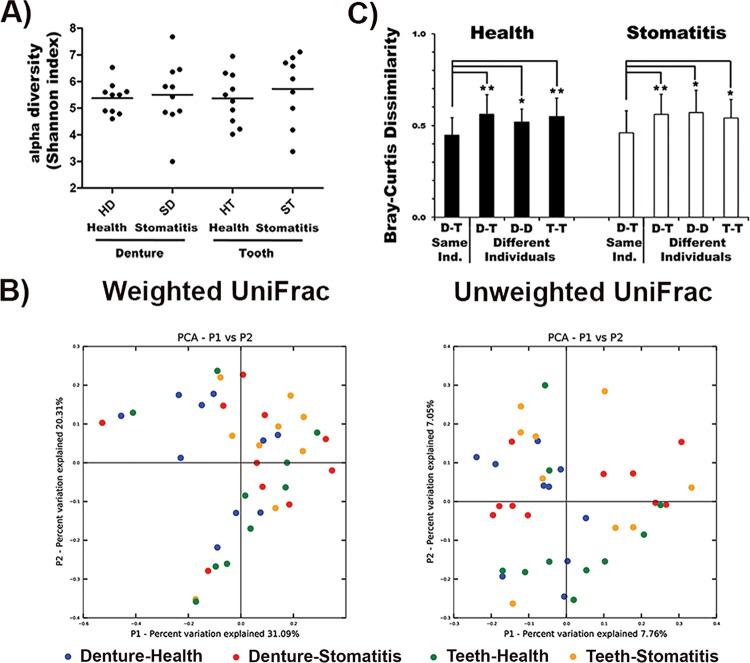
Microbial community structure analysis. (A) Alpha diversity (Shannon index) of the microbial community between dentures and teeth in health and disease, (B) Beta diversity (principal-coordinate analysis [PCoA]) of the microbial communities colonizing dentures (blue) or remaining teeth (green) of healthy individuals and stomatitis patients (red, dentures; yellow, remaining teeth), respectively. (C) Phylotype co-occurrence (calculated as Bray-Curtis dissimilarity) in denture and remaining-tooth samples from healthy individuals (black bars) and stomatitis patients (white bars). The phylotypes colonizing dentures (D) and teeth (T) were compared within the same individuals (D-T Same Ind.), between different individuals (D-T Different Individuals), and between denture (D-T Different Individuals) and tooth (T-T Different Individuals) samples from different individuals. *, *P* < 0.05; **, *P* < 0.001.

The 16S rRNA gene sequences were processed as described in Materials and Methods. Twenty-six different genera representing a total of 136 different species/phylotypes were present in at least two subjects at a relative abundance of >1% (Fig. 1). Microbial community evenness and richness (alpha diversity) at the genus level were very similar between dentures and remaining natural teeth in health, as well as disease (Fig. 2A). The microbial community structure (beta diversity) was not statistically significantly different among the four different groups (dentures-health; dentures-stomatitis; remaining teeth-health; remaining teeth-stomatitis), regardless of which aspect was examined (Fig. 2B; see Fig. S2 in the supplemental material). We then compared the individual taxa present in the samples derived from the dentures and natural teeth of the same individual with denture-tooth, denture-denture, and tooth-tooth combinations from different individuals by using Bray-Curtis dissimilarity, which, unlike UniFrac, does not include phylogenetic relatedness between community members into account. The average phylotype co-occurrence in the corresponding denture-tooth samples derived from the same study participants was significantly greater (lower Bray-Curtis index) than in the different sample combinations derived from different individuals (Fig. 2C). Thus, microbial colonization of different surfaces within an individual appears to be more similar than the same surface (dentures or remaining teeth) between individuals.

10.1128/mSphere.00215-16.2 Figure S2  Beta diversity (principal-coordinate analysis) of the microbial communities colonizing healthy individuals (green) and stomatitis patients (red) irrespective of the surface (A) and colonizing dentures (blue) and remaining teeth (black) irrespective of denture-related oral health status (B). Download Figure S2, TIF file, 0.4 MB. Copyright © 2016 Shi et al. 2016 Shi et al. This content is distributed under the terms of the Creative Commons Attribution 4.0 International license.

Of the 26 genera identified (see Fig. S3A in the supplemental material), 25 were found in dentures, while 24 colonized teeth (see Fig. S3B) at the above cutoff criteria. *Eikenella* and *Abiotrophia* in significant numbers were absent from remaining teeth, while the genus *Gemella* was largely lacking on dentures. The genus *Actinomyces* was most pervasive on both surfaces, dentures and remaining teeth, followed by *Streptococcus*, *Veillonella*, *Capnocytophaga*, *Neisseria*, *Prevotella*, and *Corynebacterium*, all of which were identified in more than half of the samples tested, independently of the surface or health status. Other genera meeting the above cutoff criteria included *Rothia*, “*Candidatus* TM7” (“*Candidatus* G-1”), *Leptotrichia*, *Porphyromonas*, *Selenomonas*, *Campylobacter*, *Lautropia*, *Granulicatella*, *Haemophilus*, *Kingella*, *Fusobacterium*, *Bacteroidales* (“*Candidatus* G-5”), “*Candidatus* TM7” (“*Candidatus* G-5”), *Actinobaculum*, *Tannerella*, and *Clostridiales* (“*Candidatus* F-2,” “*Candidatus* G-5”), as well as *Gemella*, *Eikenella*, and *Abiotrophia*. None of the apparent differences in prevalence or relative abundance were significant.

10.1128/mSphere.00215-16.3 Figure S3  Genera present at a >1% relative abundance in at least two patient samples were included in the analysis. Shown are the prevalence (a) and average relative abundance ± SEM (b) of all samples (medium gray bars) (A), differentiated into dentures (dark gray bars) and teeth (light gray bars) (B), and in health (black bars) and stomatitis (white bars) (C). *, *P* < 0.05. Download Figure S3, TIF file, 2.5 MB. Copyright © 2016 Shi et al. 2016 Shi et al. This content is distributed under the terms of the Creative Commons Attribution 4.0 International license.

Next, we performed a similar analysis comparing samples derived from healthy subjects with those obtained from patients with stomatitis regardless of the surface they originated from (see Fig. S3C). The microbiomes of healthy subjects contained only 22 of the 26 genera included in the analysis. The missing genera were *Fusobacterium*, *Tannerella*, “*Candidatus* TM7” (“*Candidatus* G-5”), and *Eikenella*. The microbial communities isolated from stomatitis patients contained all of the genera, with the exception of *Gemella*. The only genus that exhibited significant disparities (*P* = 0.026) in relative abundance between health and disease independently of the surface was *Fusobacterium* (see Fig. S3C and S4A).

10.1128/mSphere.00215-16.4 Figure S4  Health and disease status and surface-dependent differential colonization at the genus and phylotype levels. Shown are the mean relative abundances of *Porphyromonas* sp. strain HOT-279, *P. gingivalis* and other disease-related *Porphyromonas* bacteria (*P. cantoniae*, *P. endodontalis*, and *Porphyromonas* sp. strain HOT-275 combined) (A) and *Campylobacter showae*, *Capnocytophaga* sp. strain HOT-329, and *P. melaninogenica* (B). Samples are differentiated into health (black bars), disease (white bars), dentures (dark gray bars), teeth (light gray bars), dentures in health (blue bars), dentures from stomatitis patients (red bars), teeth in health (green bars), and teeth from stomatitis patients (yellow bars). *, *P* < 0.05. Download Figure S4, TIF file, 1.3 MB. Copyright © 2016 Shi et al. 2016 Shi et al. This content is distributed under the terms of the Creative Commons Attribution 4.0 International license.

**FIG 3:**
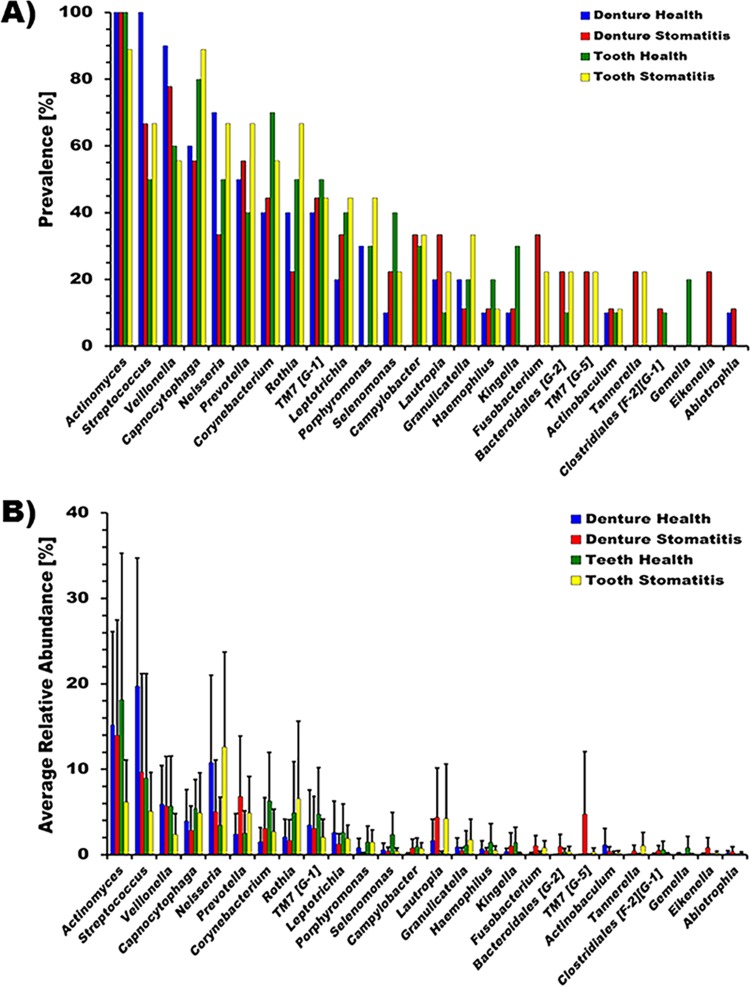
Genus prevalence and average relative abundance of denture- and tooth-associated microbiomes in health and stomatitis. Genera present at a >1% relative abundance in at least two patient samples were included in the analysis. Shown are the prevalence (A) and average relative abundance ± SEM (B) on dentures in health (blue bars), on dentures of stomatitis patients (red bars), on teeth in health (green bars), and on teeth in stomatitis patients (yellow bars). Numbers preceded by the letters G and F refer to as-yet-unnamed genera and families, respectively, within the phyla and orders indicated.

In additional comparisons, we further divided the samples into those obtained from the dentures and teeth of healthy individuals and patients with denture stomatitis (Fig. 3). The microbiome of dentures from stomatitis patients was most diverse and contained 24 different prevalent genera, while only 18 genera were found on the dentures of healthy individual. In each case, health and disease, the remaining teeth contained 21, albeit not identical, genera. Several of the genera that were present at a lower prevalence were predominantly detected in particular conditions. *Kingella* and *Gemella* mainly colonized the teeth of healthy denture wearers, while *Bacteroidales* (“*Candidatus* G-5”) was absent. *Eikenella* was limited to diseased dentures. The genus *Porphyromonas* met the 1% relative abundance cutoff for all conditions except diseased dentures. In *Fusobacterium*, *Tannerella*, and “*Candidatus* TM7” (“*Candidatus* G-5”), which were found predominantly in patients with disease, no surface-dependent differences were detected.

### Specific species/phylotypes exhibit distinct health- and disease-associated surface colonization of teeth and dentures.

**FIG 4:**
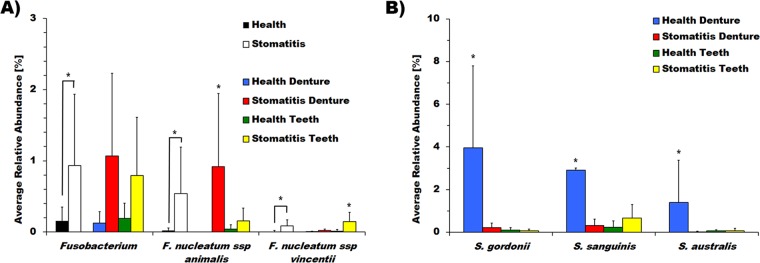
Differential colonization of dentures or teeth in health and disease at the genus and phylotype levels. Shown is the mean relative abundance of the genus *Fusobacterium*, as well as *F. nucleatum* subsp. *animalis* and *vincentii* (A), *S. gordonii*, *S. sanguinis*, and *S. australis* (B). Samples are differentiated into health (black bars), disease (white bars), dentures in health (blue bars), dentures from stomatitis patients (red bars), teeth in health (green bars), and teeth from stomatitis patients (yellow bars). *, *P* < 0.05.

Further detailed species**/**phylotype level analysis was performed for genera that occurred in at least 10% of the samples to rule out the influence of phylotypes that occur in only one or two samples with very high relative abundance and thus are not representative of the condition. This analysis revealed that *Fusobacterium nucleatum* subsp. *animalis* was found almost exclusively on the dentures of stomatitis patients (*P* = 0.016). *F. nucleatum* subsp. *vincentii*, in contrast, was found mostly on the remaining teeth of stomatitis patients (*P* = 0.0097) (Fig. 4A), while *F. nucleatum* subsp. *polymorphum* and *F. periodonticum* were not significantly different on either surface in health or disease (data not shown). Even though *Streptococcus* did not stand out as a genus, several species, including *Streptococcus gordonii* (*P* = 0.012), *S. sanguinis* (*P* = 0.049), and *S. australis* (*P* = 0.0215), colonized the dentures of healthy denture wearers at significantly higher levels (Fig. 4B). Interestingly, *Porphyromonas* sp. strain HOT-279, a phylotype of a genus often associated with oral diseases (26), was significantly more abundant in health (*P* = 0.048) (see Fig. S4A). Most of the other representatives of this genus were strongly associated with teeth in stomatitis patients, albeit at a relatively low abundance. Surface-dependent differences irrespective of health/disease status were observed for *Campylobacter showae* (*P* = 0.0173), *Capnocytophaga* sp. strain HOT-329 (*P* = 0.045), and *P. melaninogenica* (*P* = 0.026), with the first two being present predominantly on teeth and the latter one being significantly more abundant on denture surfaces (see Fig. S4B).

In addition, each denture sample was evaluated for the presence of *Candida* via PCR. In the stomatitis patient group, *Candida* was detected in 90% of the samples compared to 50% of those from the healthy group (Table 1). Several bacterial species, including *Atopobium parvulum*, *Lachnospiraceae* sp. strain HOT-097, and *Veillonella atypica*, were present mainly in denture samples containing *Candida*, while *Leptotrichia* sp. strain HOT-212, with the exception of one patient, was present only in samples without *Candida*.

**TABLE 1:** Evaluation of denture samples for the presence of *Candida* via PCR and co-occurrence with bacterial species/phylotypes

Microorganism	Healthy patients										Stomatitis patients								
	HD1	HD2	HD3	HD4	HD5	HD6	HD7	HD8	HD9	HD10	SD1	SD2	SD3	SD4	SD5	SD6	SD8	SD9	SD10
*Candida*	+^a^	−	−	+	−	−	+	+	−	+	+	+	+	+	+	+	+	−	+
*Atopobium parvulum*	+	−	−	+	−	−	−	−	−	+	+	+	−	+	+	+	+	−	−
*Lachnospiraceae* sp. strain HOT-097	+	−	−	−	−	−	−	−	−	+	+	+	−	+	−	−	+	−	+
*Leptotrichia* sp. strain HOT-212	+	+	−	−	+	+	−	−	+	−	−	−	−	−	−	−	−	+	−
*Veillonella atypica*	+	−	−	+	−	+	−	−	−	+	−	+	−	−	+	−	−	−	+

a Symbols: +, presence of *Candida* in sample; −, absence of *Candida* in sample (as determined by PCR). The presence or absence of bacterial species was derived from the 16S rRNA gene sequencing data.

## DISCUSSION

Denture stomatitis has largely been studied in the context of fungal infections, and even though there was a suggestion decades ago by Koopmans and coworkers “to pay more attention to the bacterial population in denture-induced stomatitis instead of focusing only on *Candida albicans*” (11), few studies have done so since. A detailed clone library analysis (10) provided a first glimpse into the diversity of bacterial phylotypes associated with dentures in health and disease, while a recent predominantly class and phylum level study (12) introduced modern sequencing approaches to the field. The data presented here comprise the first comprehensive genus and species level analysis of the bacteria residing in biofilms on the dentures and remaining teeth of healthy patients and those with stomatitis. Importantly, comparison of samples derived from the dentures and remaining teeth of the same individuals allowed us to analyze the possible mutual influence of the bacterial communities colonizing these different surfaces in health and disease.

Our initial genus level analysis revealed that, unlike the distinct health- and disease-associated microbiota reported previously for other oral diseases such as periodontitis (27–30), the bacterial communities residing on dentures and remaining teeth in health and disease are rather similar to each other (Fig. 1). This lack of differences in bacterial community composition was also reflected by the respective alpha and beta diversities (Fig. 2A and B; see Fig. S2). Consistent with previous findings of large individual dependent variations of the microbiome (29, 31, 32), we found that the phylotype composition of bacterial communities growing on dentures and those derived from remaining teeth were significantly more similar to each other (lower Bray-Curtis dissimilarity index) in samples derived from the same individual than in unrelated denture and tooth samples from different individuals (Fig. 2C). This is also reflected in our observation that only three species**/**phylotypes displayed significant differential surface colonization (see Fig. S4B). Considering this apparent strong mutual influence of bacteria colonizing dentures and teeth in the same individual, the health and integrity of remaining teeth could comprise an important factor in the mucosal health of denture wearers beyond their role in anchoring restorations and maintaining bone integrity. Similarly, the denture-associated oral mucosal health status could play a critical role in conserving remaining teeth.

Furthermore, the bacterial phylotype compositions present in biofilms collected from the same surface (dentures or remaining teeth) of different patients were significantly less similar to each other than to the communities identified on the matched denture-teeth samples in the same patients (Fig. 2C). This is interesting, since biofilm formation is thought to be rather surface dependent (7), and while overlap exists, different natural surfaces within the oral cavity are colonized by distinct communities (25). Our findings indicate that individual-specific factors can be more dominant determinants of the oral bacterial biofilm community composition than surfaces. One important host factor involved in this phenomenon could be saliva, which coats available natural, as well as artificial, oral surfaces with a so-called pellicle (33). Saliva can display large variability between individuals (34, 35) and provides important adhesion proteins for bacterial attachment (36). In addition, our discovery that intrapatient factors could be stronger determinants of bacterial biofilm community composition than different surfaces emphasizes that grouping and pooling of samples from different people can influence analysis outcome.

Previous studies analyzing the bacteria colonizing dentures in health and disease are not conclusive. Similar to our results, a recent sequencing study (12) and older culture-based approaches (11, 16, 24) found no difference in the apparent microbial composition between healthy and stomatitis patients and noted only that the amount of plaque buildup is significantly greater in stomatitis patients. In contrast, a clone library-based culture-independent study (10) reported that the microbiota of biofilms colonizing dentures in health and disease are distinct. Furthermore, unlike our results detailed above, a recently published sequencing study (12) described a significant difference between the bacterial community compositions found on denture surfaces and those on remaining teeth. The apparent discrepancies between our findings and previous 16S rRNA gene-based sequencing studies can be due to many factors, ranging from geographical differences between patient populations to sample collection, sequencing parameters (choice of 16S rRNA target region, the sequencing platform used, available read length, and sequencing depth, among others), or DNA extraction and PCR protocols (37).

Not surprisingly, we found *Actinomyces*, *Capnocytophaga*, *Streptococcus*, *Veillonella*, and *Neisseria* to be most prevalent and abundant genera, independently of the surface or health/disease status, in all of our samples (Fig. 3; see Fig. S3). These genera are among the most predominant in the oral cavity and have been identified as major denture colonizers in previous culture-based and culture-independent studies (10–12, 16). Especially the genera *Actinomyces* and *Streptococcus* are considered early colonizers of the oral cavity that readily attach to available surfaces, as well as each other (38, 39). They enable surface colonization of other microbial species, including *Capnocytophaga* and *Neisseria*, via physical binding, as well as metabolic interactions such as the metabolic interdependence between *Veillonella* and *Streptococcus* species (40, 41). Other prevalent genera present in the samples analyzed in our study include *Corynebacterium*, *Rothia*, several genera of “*Candidatus* TM7,” and *Fusobacterium*. Most previous studies comparing denture plaque in health and disease did not identify these genera (10–12), even though they were found to colonize denture teeth in a checkerboard study comparing natural tooth and denture colonization patterns (15). Consistent with earlier studies (10, 12, 23, 24, 42, 43), *Candida* was not limited to denture stomatitis samples, with fewer of the healthy samples being positive (Table 1). While previous class level analysis indicated that *Candida* colonization was positively correlated with lactobacilli and negatively correlated with *Fusobacteria*, this was not the case for the samples analyzed here. In our study, we observed a possible positive correlation for *A. parvulum*, *Lachnospiraceae* sp. strain HOT-097 (“*Candidatus* G-4”), *Veillonella atypica*, while *Leptotrichia* sp. strain HOT-212 was not present in samples containing *Candida*, with the exception of one healthy patient. Since little is known about the interaction between these species and *Candida*, further study is needed to confirm the relevance of this observation.

Despite the similarities on the biofilm community level, individual genera and species were significantly different in their occurrence on specific surfaces and/or the denture-related health/disease status of the patient. All members of the genus *Fusobacterium* had very low colonization rates on healthy dentures and health in general (Fig. 4A), even though the species *Streptococcus gordonii* and *S. sanguinis* that fusobacteria are known to attach to (44, 45) were present in significantly elevated numbers under this condition (Fig. 4B). These findings indicate that in the complex biofilm environment of the oral cavity, factors beyond the ability to bind to each other play important roles in microbial community dynamics and composition. Among the different *Fusobacterium* species and subspecies, *F. nucleatum* subsp. *polymorphum*, *F. nucleatum* subsp. *vincentii*, and *F. periodonticum* have previously been observed to increase more on natural teeth than on denture teeth (15). In our study, they exhibited a higher relative abundance on natural teeth in disease; however, this difference was only significant for *F. nucleatum* subsp. *vincentii* (Fig. 4A). Surprisingly, the colonization pattern of *F. nucleatum* subsp. *animalis* was completely different from that of all of the other fusobacterial species and subspecies identified, with a striking almost exclusive colonization of denture surfaces in stomatitis patients (Fig. 4A). This particular subspecies of *F. nucleatum* has been identified as an etiological agent in a case report connecting the oral microbiota with stillbirth (46), documented to be more prevalent in subgingival plaque and early periodontitis (47), as well as experimental gingivitis (48). Furthermore, *F. nucleatum* subsp. *animalis* is part of the microbial signature in early detection of colorectal cancer (49) and the only fusobacterial species**/**subspecies found to overlap between the microorganisms isolated from the periodontal pocket and the atheromatous plaque in cardiac disease patients (50). Our finding of a strong association of *F. nucleatum* subsp. *animalis* with the inflammatory mucosal condition stomatitis in combination with the above findings by other research groups suggests that this *F. nucleatum* subspecies may be more pathogenic than others.

In addition to the prominent difference in *F. nucleatum* subsp. *animalis* distribution, which could be relevant for stomatitis etiology, other microorganisms displayed disparate surface- and/or health status-dependent colonization patterns. As already mentioned above, certain *Streptococcus* species displayed a significantly higher prevalence and abundance on dentures in healthy denture wearers than under all other conditions (Fig. 4B). Among these, *S. sanguinis* and *S. gordonii* have previously been associated with oral health (51–53), and a recent study revealed a distinct, species-specific distribution of streptococci on the natural oral surfaces of healthy subjects (25). Additional evidence for the importance of analysis beyond the genus or phylum level provides the colonization pattern of the genera *Fusobacterium* and *Porphyromonas*. While their presence correlated strongly with health and disease independently of the surface when analyzed on the genus level (Fig. 4A; see Fig. S4A), detailed species**/**phylotype level analysis yielded a more differentiated picture. As already discussed above, the different representatives of the genus *Fusobacterium* present in our samples displayed a distinct surface and denture health status-dependent distribution (Fig. 4A). Our finding that *Porphyromonas*, a genus typically associated with disease (26), exhibited a significant surface-independent health association (see Fig. S4A) provides an example of how the lack of taxonomic resolution could influence results and data interpretation. Phylotype level examination revealed that the genus *Porphyromonas* was predominantly represented in our samples by *Porphyromonas* sp. strain HOT-279, a phylotype that is abundant in healthy human subjects who participated in the Human Microbiome Project (25, 54). Additional, less abundant representatives of *Porphyromonas* (*P. gingivalis*, *P. endodontalis*, *P. catoniae*, and *Porphyromonas* sp. strain HOT-275) exhibited the “typical” disease association of this genus, as they were significantly correlated with the remaining dentition of stomatitis patients (see Fig. S4A). Since these distinct health- and disease-associated distribution patterns of individual representatives of certain genera are apparent only on the phylotype/species level, genus level microbiome analysis is not always sufficient to provide a comprehensive picture of the relevant players and higher-resolution analyses could be critical for in-depth understanding of the oral microbiome in health and disease.

In conclusion, our study suggests that the bacterial microbiota on dentures is highly similar in health and disease on the broader community level. This was also observed for dentures and remaining teeth independently of health status, especially in samples derived from the same individuals. The phylotype composition of the bacterial communities colonizing the dentures and remaining teeth of the same individuals are largely reflective of each other, indicating a possible mutual influence of denture health status on the dentition and vice versa. The observed lack of distinct microbiota is consistent with most previous reports that in denture-associated oral diseases, the overall microbial load may have a greater impact on stomatitis development than the actual microbial composition of the mucosa-facing denture plaque. Despite these overall similarities, we were able to identify distinct species such as *F. nucleatum* subsp. *animalis* and several species of *Streptococcus* that were strongly associated with diseased and healthy denture samples, respectively. Our findings that significant differences in colonization were observed predominantly on the phylotype**/**species level highlight the importance of species/phylotype or even oligotype level analysis (25).

## MATERIALS AND METHODS

### Subject population and sample collection.

Twenty adult denture-wearing volunteers with a minimum of four remaining teeth and one complete denture were recruited for this study under Institutional Review Board no. 2012-0004 to West China University (Chengdu, China). Ten individuals were healthy denture wearers, and 10 were patients with denture-associated stomatitis according to published guideline for diagnosis of denture stomatitis (55). The group of healthy denture wearers consisted of five women and five men with a mean age of 69.8 ± 4.7 years. Similarly, the group of stomatitis patients included five women and five men with a mean age of 61.1 ± 12.0 years. The study participants did not have any other active oral diseases such as caries or periodontitis. Eligible individuals were systemically healthy and not taking any prescription or nonprescription medication for at least 6 months. Additionally, the study participants had not used any biocide-containing toothpaste or denture cleanser for the past 6 months. Informed consent was obtained prior to sample collection and signed by study participants, as well as the clinicians and study personnel performing oral health evaluations, sample collection, and processing.

Study participants were asked to wear their dentures for at least 3 h and refrain from eating, drinking, and tooth or denture cleaning prior to plaque sampling. Plaque samples were collected by a trained dentist from the parts of the pink acrylic denture surface that was in contact with the oral mucosal surface by applying sterile toothpicks with a circular motion. Using a similar circular motion, sterile toothpicks were also employed to obtain supragingival plaque from the remaining teeth. Care was taken to collect from the buccal surfaces of teeth that were not in direct contact with denture surfaces. Individual denture and tooth plaque samples, as well as control toothpicks, were placed into separate microcentrifuge tubes containing 0.5 ml of oxygen-reduced phosphate-buffered saline and immediately stored at −20°C until DNA extraction.

### DNA extraction and sequencing.

Genomic DNA was isolated from the collected plaque samples and the corresponding sterile toothpick controls with the DNeasy Blood and Tissue kit (Qiagen Inc., United States) as previously described (56) with the addition of bead beating for maximal cell lysis (29). DNA quality and quantity were determined with a NanoDrop 2000 spectrophotometer (Thermo, United States). After genomic DNA extraction and quantification, DNA concentrations of the samples were normalized to 2 to 6 ng/μl and PCR amplification was performed according to the protocol developed by the Human Microbiome Project for sequencing on the 454 FLX titanium platform (54). Briefly, hypervariable regions V1 to V3 of the 16S rRNA genes were amplified from purified genomic DNA with primers 27F (V1 primer, 5′AGAGTTTGATCCTGGCTCAG3′) and 534R (V3 primer, 5′ATTACCGCGGCTGCTGG3′) fitted with individual barcodes and the A adapter sequence (5′CCATCTCATCCCTGCGTGTCTCCGACTCAG3′) for the 534R primer and the B adapter (5′CCTATCCCCTGTGTGCCTTGGCAGTCTCAG3′) for the 27F primer and pooled for sequencing. The 16S rRNA gene amplicon libraries were constructed and 454 pyrosequencing was performed at the J. Craig Venter Institute Joint Technology Center.

### Microbial taxonomic composition analysis.

The 454 pyrosequencing data were demultiplexed into the respective samples on the basis of the individual barcodes of each sample. After the bar codes were trimmed, a data cleaning process was applied to all of the samples with an in-house Perl program. Briefly, low-quality sequences containing bases with a Phred quality value of <20, were trimmed off the read ends. Sequences with a final read length of <300 bp or with ≥3% uncertain bases were removed. Suspected chimeras were identified and removed with ChimeraSlayer (57). The 16S rRNA sequences were clustered into operational taxonomic units (OTUs) at a 97% sequence similarity level with QIIME (58). OTUs were then annotated with taxonomic assignment by comparing the representative sequence of each OTU to the Human Oral Microbial Database references (16S rRNA gene RefSeq version 11.0) (59) with BLAST on the basis of the best match at >97% nucleotide sequence identity over at least 95% of the length of the query (56). The microbial community evenness and richness were measured by alpha diversity, and the similarity between individual microbial communities was measured by beta diversity. Alpha diversity (Shannon index), beta diversity (weighted and unweighted UniFrac), principal-coordinate analysis, and sequencing depth assessment by rarefaction analysis were calculated or performed with QIIME (58) at the OTU level. Genera present in at least two subjects at a relative abundance of >1% were included in further genus and species**/**phylotype level analysis. The taxonomic composition of each sample was summarized at the genus and species levels.

### Detection of *Candida* via PCR.

The presence or absence of *Candida* in the samples collected from dentures was assessed with universal primers ITS1 (5′ CTTGTTATTTAGAGGAAGTAA 3′) and ITS2 (5′ GCTGCGTTCTTCATCATGC 3′) (60) under the following cycling conditions: 94°C for 11 min, followed by 35 cycles of 94°C for 30 s, 50°C for 30 s, and 72°C for 30 s with a final 30-min extension at 72°C.

### Statistical analysis.

Data are represented as mean ± the standard error of the mean (SEM) unless otherwise indicated. All data sets were examined for their distribution properties with the Shapiro-Wilk test prior to analysis. Statistical testing of differences in relative abundance of genus and species distribution between the different sample groups was tested with the Mann-Whitney U test for pairwise comparisons (dentures versus teeth; health versus stomatitis) and the Kruskal-Wallis test for comparison of multiple groups (dentures versus teeth in health and disease). Differences in the prevalence of genera and phylotypes between multiple groups were evaluated with Fisher’s exact test. Bray-Curtis dissimilarity was employed to evaluate the copresence of bacterial phylotypes on dentures and teeth derived from the same individuals compared to dentures and teeth from different individuals and only dentures or only teeth from different individuals. The Bray-Curtis dissimilarity results were further analyzed with a two-tailed unpaired *t* test and analysis of variance. All statistics were performed with the respective features in R Studio, Prism, and Excel, while the nonparametric multivariate analysis anosim in mothur (61) was used to test whether the microbiome similarities within groups are statistically significantly different from the similarities between groups. The *P* values were adjusted for multiple testing of microbial taxa with p.adjust in R by using the false-discovery rate (62).

### Availability of data.

The data set supporting the conclusions of this article are available at the NCBI BioProject under accession number PRJNA292354.
