# Supplementary figures and images for: The Denture-Associated Oral Microbiome in Health and Stomatitis
Source: mSphere. 2016 Dec 28;1(6):e00215-16. doi: 10.1128/mSphere.00215-16 (PMC5196032; doi:10.1128/mSphere.00215-16)

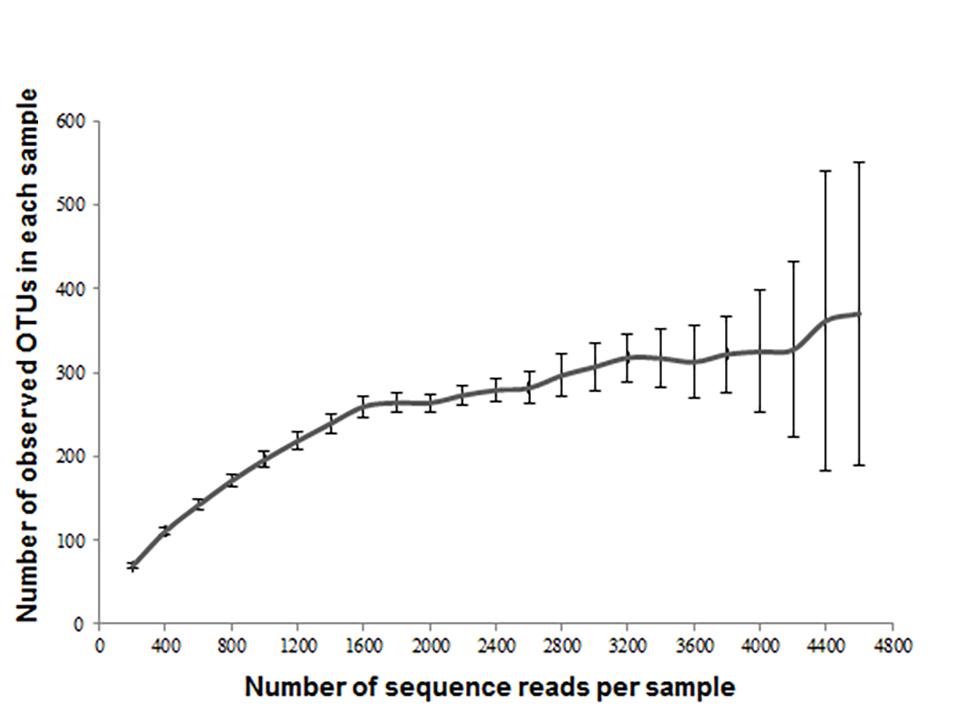

Supplement: Figure S1 [file sph006162215sf1.tif]

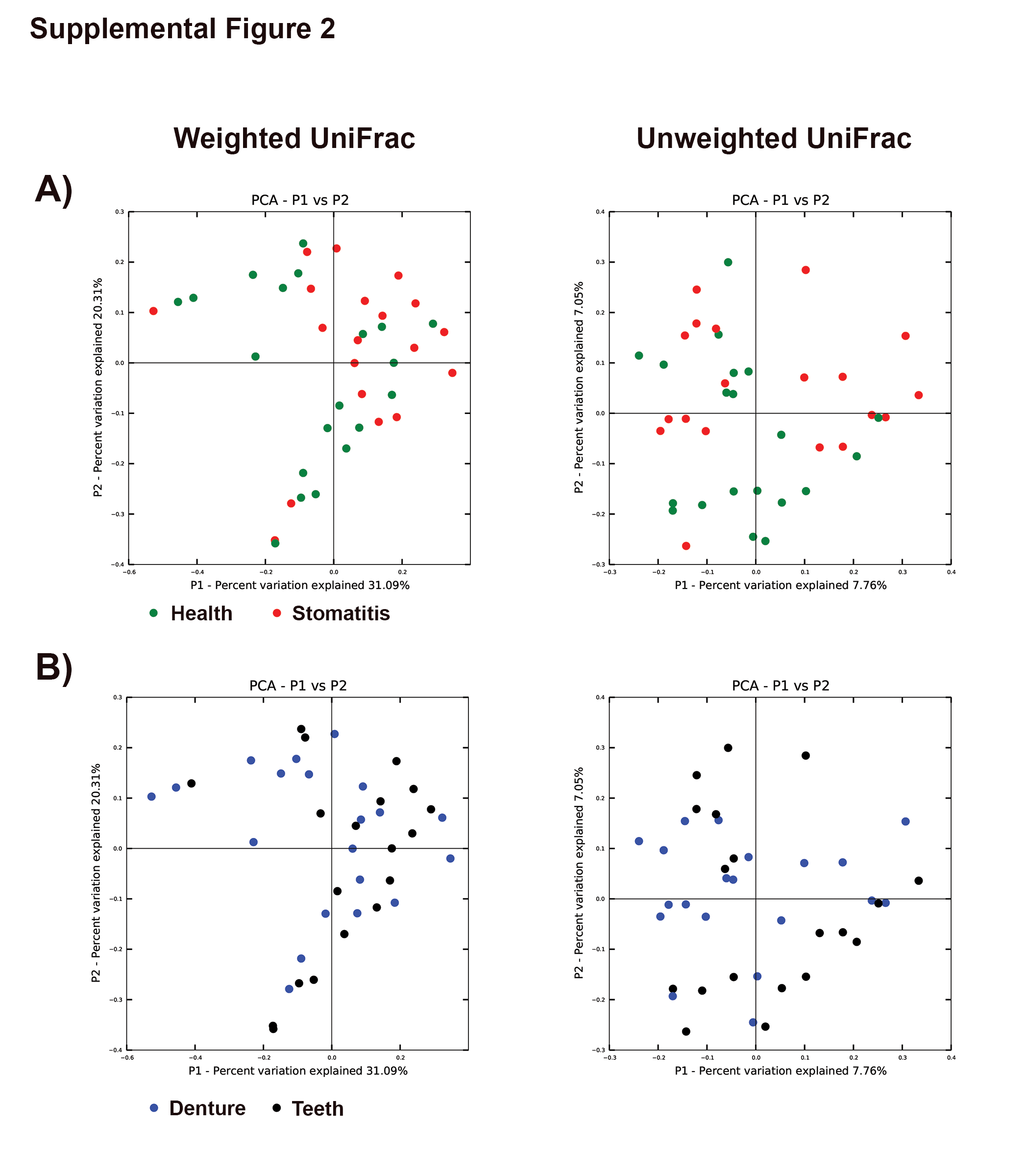

Supplement: Figure S2 [file sph006162215sf2.tif]

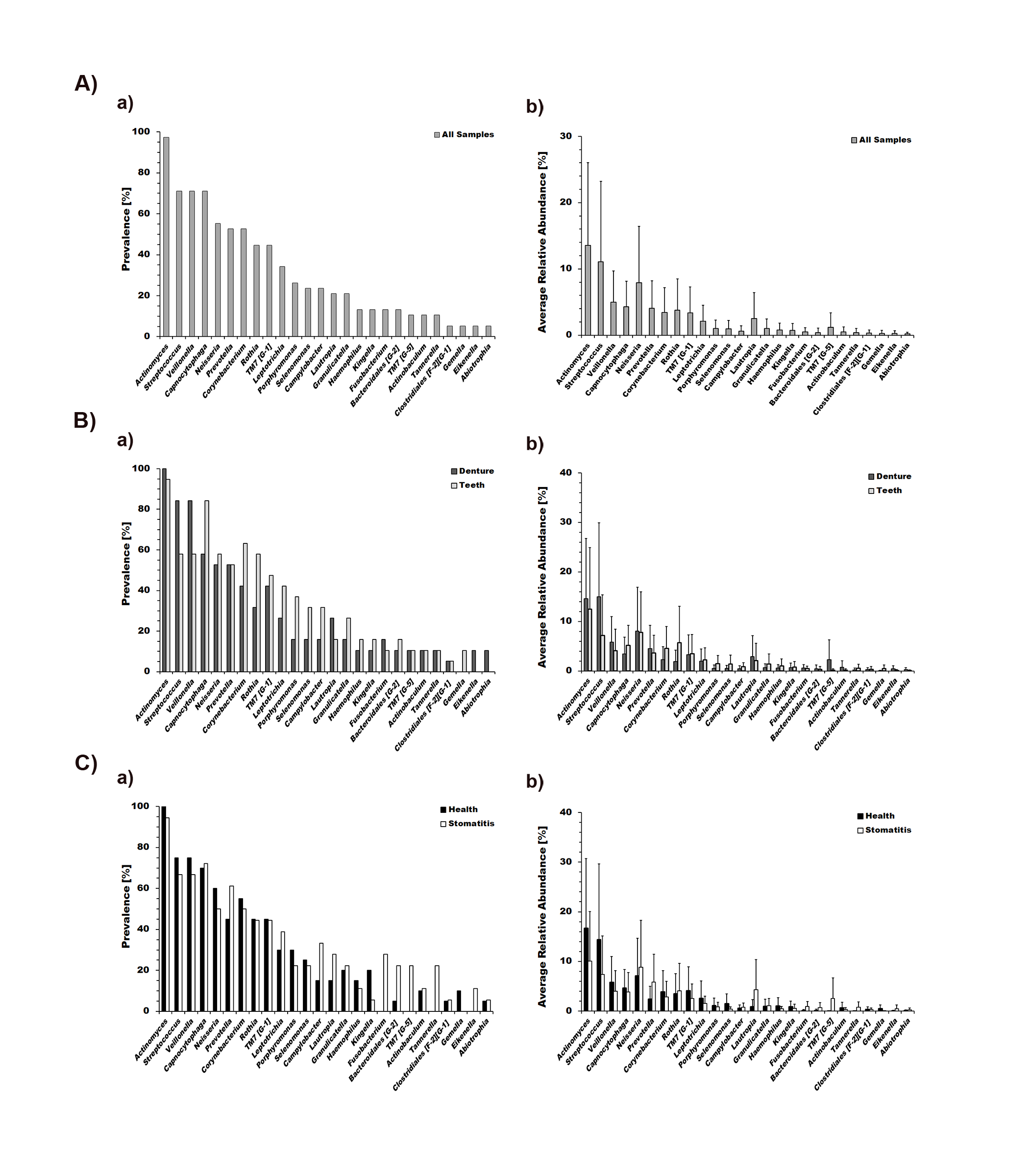

Supplement: Figure S3 [file sph006162215sf3.tif]

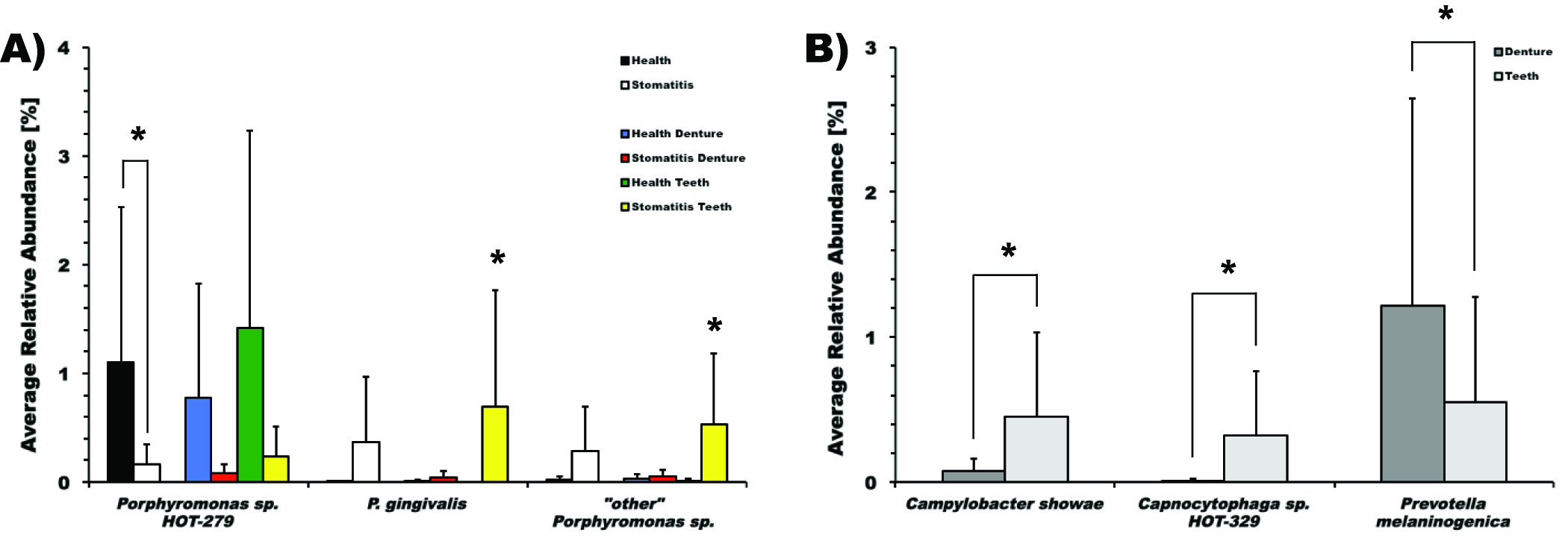

Supplement: Figure S4 [file sph006162215sf4.tif]
